# Effects of six weeks high-intensity interval training and resistance training in adults with obesity and sleep related breathing disorders

**DOI:** 10.5935/1984-0063.20200076

**Published:** 2021

**Authors:** Khomkrip Longlalerng, Anucha Nakeaw, Asmu-e Charawae, Powpachara Reantong, Usamawee Prangyim, Nutjaree Jeenduang

**Affiliations:** 1 Walailak University, School of Allied Health Science, Department of Physical Therapy - Thasala - Nakhon Si Thammarat - Thailand.; 2 Walailak University, School of Allied Health Science, Department of Medical Technology - Thasala - Nakhon Si Thammarat - Thailand.

**Keywords:** High-intensity Interval Training, Resistance Training, Sleep-related Breathing Disorders, Obesity

## Abstract

**Introduction:**

The effects of high-intensity interval training (HIIT) combined with resistance training (RT) in adults with obesity and sleep-related breathing disorders (SRBDs) is limited.

**Objective:**

This study aimed to examine the effects of HIIT combined with RT on subjective sleep disorders in adults with obesity and SRBDs.

**Material and Methods:**

This study was a pre- and post-test design. Seventeen adults with obesity and SRBDs were recruited into the study. They received 24 minutes of HIIT and 30 minutes of RT, 3 times/week for 6 weeks. The Epworth sleepiness scale (daytime sleepiness), Berlin questionnaire (snoring and daytime sleepiness category), estimated maximum oxygen consumption (VO_2_max), muscle strength using 1-repetition maximum, anthropometric variables, and blood biomarkers were examined at baseline and after 6 weeks of training.

**Results:**

The Epworth sleepiness scale, Berlin questionnaire (daytime sleepiness category), and the number of risks associated with sleep apnea using the Berlin questionnaire were significantly decreased after 6 weeks of training (all p<0.01). The estimated VO_2_max and muscle strength were significantly increased at Week 6 (all *p*<0.05). Body weight, body mass index, % body fat, and hip circumference were significantly decreased at Week 6 (all *p*<0.05). No significant changes were observed in blood biomarkers, except for fasting blood glucose (p<0.01).

**Conclusion:**

Six weeks of HIIT combined with RT has beneficial effects on subjective sleep disorders, estimated VO_2_max, muscle strength, and most anthropometric variables in adults with obesity and SRBDs.

## INTRODUCTION

Sleep-related breathing disorders (SRBDs) are a wide spectrum of breathing difficulties during sleep in adults^1^. Its clinical characteristics range from habitual snoring, fragmented sleep to intermittent hypoxia, and obstructive sleep apnea (OSA)^1,2,3^. The most severe form of SRBDs is OSA, which ranges from 3.7-93.3% prevalence in Asian adults depending on age, gender, body composition, and comorbidities^4^. SRBDs are related to functional decline, excessive daytime sleepiness (EDS), cognitive impairment, mood disturbance, medical comorbidities, and obesity^3,5^. There is evidence to support the suggestion that SRBDs are a preliminary condition of endothelial dysfunction leading to atherosclerosis and cardiovascular diseases^6^. Therefore, early detection and intervention of problems related to SRBDs are necessary to eliminate the event of fatal effects.

Conservative intervention has been established as an alternative treatment for SRBDs, such as behavioral modification, including exercise and/or combined with diet control^7,8,9,10,11,12,13^. It was found that these interventions were beneficial effects on sleep parameters^7,8,9,10,11,12,13^. Previously, a number of studies found that EDS^7,9,11^, snoring^10^, and sleep indices^9,12,13^ were improved after exercise intervention. More recently, high-intensity interval training (HIIT) has become more attractive form of exercise program because it is capable of reducing cardiovascular risk factors related to obesity and improving physical fitness^14,15^. A growing body of evidence revealed that HIIT is safe and practical to apply with many vulnerable subjects^14,15^. It has superior effectiveness over traditional moderate-intensity aerobic training in mitigating many circumstances^15^. In addition, resistance training (RT) is considered as adjuvant therapy for weight management in obese adults^16^. There was a study showing that RT could provide a greater advantage if combined with aerobic exercise for obese adults^17^.

Nevertheless, there are limited studies examining the combined effects of aerobic exercise in terms of a HIIT program with RT for adults with obesity and SRBDs. Thus, this study aimed to examines the effects of HIIT and RT on the subjective sleep disorders of adults with obesity and SRBDs. We hypothesized that six weeks of this combined intervention would significantly improve the subjective sleep disorders of adults with obesity and SRBDs.

## MATERIAL AND METHODS

### Study population

Male and female adults aged 20-53 years were recruited into the study. They were asked to complete two sleep questionnaire forms, including the Epworth sleepiness scale and Berlin questionnaire for first time screening. The study was conducted at Thasala District, Nakhon Si Thammarat Province, Thailand, from February to April 2020. The inclusion criteria for participants consisted of: 1) those who were suspected to have SRBDs from the questionnaire forms (the Epworth sleepiness scale ≥10 or snoring item of the Berlin questionnaire ≥2); and 2) those who were classified as a person with obesity based on their BMI using the criteria of the International Obesity Task Force World Health Organization Western Pacific Region (BMI≥25kg/m^2^)^18^. The exclusion criteria for participants consisted of: 1) those who have received other treatments related to SRBDs or have undergone behavioral modification programs; and 2) those who have underlying diseases (e.g., cardiorespiratory disorder, hypertension, diabetes mellitus, neurological disorders, or any orthopedic problems that limited exercise performance). This study was approved by the Human Research Ethics Committee of Walailak University, Nakhon Si Thammarat Province (#WUEC-20-006-01). Written informed consent was acquired from all participants (Thai Clinical Trials Registry: TCTR20200216001).

### Study design

**Figure 1:**
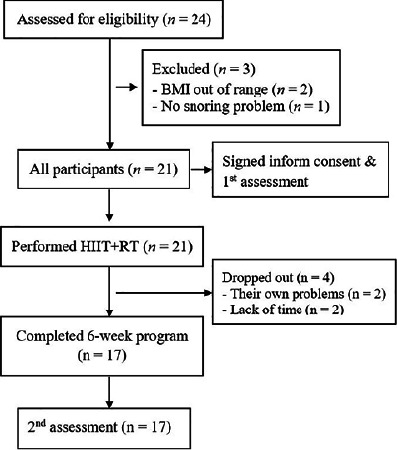
Flow chart of the study

This study was a time-series design with pre- and post-test comparisons. Participants were instructed to perform a 6-week HIIT combined with RT (n=21). They were not allowed to participate in other exercise programs during the study period. Participants were suggested to control their diet by the researchers. The primary outcome measurement were daytime sleepiness using the Epworth sleepiness scale, and OSA risk using the Berlin questionnaire, which is composed of the snoring category (items 1-5), daytime sleepiness category (items 6-8), and questions about having obesity or hypertension (items 9-10). According to the interpretation procedures based on the original version, if the respondents had a total score ≥2 in category 1 and 2, have hypertension, or are identified as obese, then they are classified as having a risk of sleep apnea. If participants had a positive risk ≥2 in three categories, then they are identified as having a high risk of OSA. The secondary outcomes included anthropometric variables [i.e., body mass index (BMI); percent body fat (%BF); neck, waist, and hip circumferences (NC, WC, HC); and waist hip ratio (W/H)], the estimated maximum oxygen consumption (estimated VO_2_max), muscle strength using 1 repetition maximum (1-RM) for the seven major muscles in the body, and blood biomarkers [triglyceride, total cholesterol (TC), high density lipoprotein cholesterol (HDL-C), low density lipoprotein cholesterol (LDL-C), and fasting blood glucose (FBG)]. All variables were examined at baseline and after 6 weeks of training. A flow chart of the study is shown in Figure 1.

### Exercise intervention program

The exercise program comprised 24 minutes of HIIT and 30 minutes of RT. All participants were asked to participate in the exercise program at the physical therapy laboratory fitness room, Walailak University, three times weekly for six weeks. The exercise program was supervised by a physiotherapist. The HIIT program was adapted based on previous studies^19,20^. The HITT intensity began at 85 maximum heart rate (MHR), and MHR was calculated using the formula (220-age) prior to participants performing the 1^st^ exercise session. The HIIT program comprised of four 3-min bouts at high intensity (>85% MHR), separated by 3 minutes of active recovery at moderate intensity (60-70% MHR). The HIIT exercise types included treadmill running, outdoor running, leg ergometer cycling, and ball activities. A polar heart rate sensor (H10) was used to monitor exercise intensity for each exercise session. Afterwards, a 30-minute RT program was performed using weight machines, dumbbells, and body work^16^. It began with 50% of 1-RM, and gradually progressed by increasing 10% every 2 weeks. The repetitions of each pose were between 10-15 times with 1-minute rest intervals, 2 sets per pose. The RT poses included the chest press machine, biceps curls, push-ups, abdominal curl-ups, lateral pulldowns, leg presses, and leg curls.

### Outcome measurements: Subjective sleep disorders

The Epworth sleepiness scale (ESS) is used to assess excessive daytime sleepiness (EDS), and has been translated into a Thai version by Banhiran et al. (2011)^21^. It is a self-administered questionnaire which takes 2-3 minutes to complete, and it is comprised of eight circumstances for the potential of dozing off or falling asleep with a rating scale of 0 to 3. The questionnaire shows an excellent internal consistency and test-retest reliability, and it can be properly used for assessing intervention efficacy. A total score of the ESS>10 is identified as a person with EDS, and there is a high risk of SRBDs. Meanwhile, the Berlin questionnaire is used to evaluate nighttime sleep issues (e.g., snoring problems), daytime sleepiness, and fatigue. It is divided into three categories including snoring, daytime sleepiness, and problems related to OSA. The Berlin questionnaire was translated into Thai by Suksakorn et al. (2014)^22^. It has satisfactory validity and reliability for evaluating a person with SRBDs. It is comprised of 10 question items and takes 5 minutes to complete. The total scores of each category ≥2 indicate a positive risk for having OSA. The 3^rd^ category is determined as a positive risk if participants have hypertension or are classified as obese. Hence, if the respondents had a positive risk ≥2 in three categories of the Berlin questionnaire, then they will be categorized as at high risk of OSA.

### Anthropometric variables

Body weight (BW), body mass index (BMI), body fat percentage (%BF), fat free mass (FFM), and muscle mass were examined using a bioelectrical impedance analyzer (Tanita SC-330, Tokyo, Japan) after 12h of overnight fasting. A tape measure was used to measure waist circumference (WC) at the umbilical level. Hip circumference (HC) was measured at the prominent part of the buttock region. Neck circumference (NC) was measured at the prominent part of the thyroid cartilage. The intra-rater reliability of WC, HC, and NC was examined in 10 healthy subjects. They were in an acceptable range of reliability (intraclass correlation coefficient >0.95, all *p*<0.01).

### Physical fitness

An estimated VO_2_max was examined using the Åstrand-Ryhming cycle ergometer test. Participants were instructed to cycle for 6 minutes following the American College of Sports Medicine guidelines^16^. The average heart rate between the last 2 minutes was used to estimate VO_2_max using a nomogram. The estimated VO_2_max (l/min) was adjusted with an age factor and transformed to relative estimated VO_2_max (ml/kg/min) using the formula:


$$\mathrm{Relative}\;{\mathrm{estimatedVO}}_{2}\max\;\left(\mathrm{ml}/\mathrm{kg}/\min\right)=\left[\left({\mathrm{estimatedVO}}_{2}\left(1/\min\right)\times 1,000\right)/\mathrm{body}\;\mathrm{weight}\left(\mathrm{kg}\right)\right]$$

Muscle strength was measured using 1-RM for all seven large major muscle groups. It was examined using dumbbells, machine stations, and body weight. The equation for estimating 1-RM (kg) is as follows:


$$1-\mathrm{RM}\left(\mathrm{kg}\right)=\mathrm{lifting}\;\mathrm{weight}\left(\mathrm{kg}\right)/{\left(1-0.02\times \mathrm{repetitions}\right)}^{23}$$

### Blood biomarkers

After 12h of overnight fasting, 9ml of blood venous samples were drawn from each participant at the antebrachial area. Total cholesterol (TC), triglyceride, high density lipoprotein cholesterol (HDL-C), and fasting blood glucose (FBG) were analyzed by the standard laboratory method. All tests were performed using an Auto Analyser A15 (BioSystems S.A, Barcelona, Spain). Low density lipoprotein cholesterol (LDL-C) was measured by the Freidewald equation.

### Sample size calculation

The sample size was calculated based on a previous study^19^ using G-power software (Version 3.1.9.4). AHI (7.5) and SD (11.6) were used to replace the G-power program (matched pairs) with an effect size of 0.65, a power of 0.8, and a significance level of 0.05. The number of samples in this study was 17 participants. To prevent a dropout percentage, we added 20 percent to account for the attrition rate. Finally, 21 participants were obtained and recruited into the study.

### Statistical analysis

The Shapiro-Wilk test was used to identify data distribution. The Paired sample t-test or the Wilcoxon signed rank test was chosen to compare the difference between baseline and after six weeks of training according to a distribution of data. The Spearman correlation coefficient was used to determine the correlation between data change of the sleep quality and study’s variable which found significant difference between the baseline and after six weeks of training. The effect size calculation and interpretation were the following as previously guidelines^24^. Descriptive data was shown as mean ± SD for continuous normal data distribution, median [Interquartile Range (IQR)] for continuous skewed data, and counts (percentages) if indicating categorical data. The Statistical Package for the Social Sciences (SPSS) Version 22.0 (SPSS Co., Ltd. Bangkok, Thailand) was used to analyze the data. The significance level was set at *p*<0.05.

## RESULTS

### Participant characteristics

Figure 1 illustrates a flowchart for participants throughout this study. Twenty-one obese adults with SRBDs were included in the study. The baseline characteristics of the participants are shown in Table 1. Most of them were not exercise regularly (<2 days/week, 71%). All participants were identified as high risk of OSA according to the Berlin questionnaire. The average exercise sessions were 16.76 sessions (93%). The average MHR was 169 beats/minute, which was approximately 91% MHR as calculated based on the average age at 34 years old. The average rating perceived exertion was 13.49. The percentage of exercise types including treadmill running, leg ergometer cycling, outdoor running, and ball activities was 72%, 16%, 10%, and 2%, respectively. At week 2, there were three participants excluded from the study due to lack of time to participate in the exercise program. At week 5, one participant requested to leave the study for personal reasons. Finally, there were 17 participants who completed the 6-week exercise program. There were two participants who exhibited musculoskeletal problems during the exercise program. However, there were no adverse events found in participants during the entire 6 weeks of training.

**Table 1:** Baseline characteristics of the participants, mean ± SD.

	All participants (n=21)	Completed exercise program (n=17)
Age (years)	34 ± 11.91	34 ± 12.39
Gender, n (%)		
Male	13 (62%)	12 (71)
Female	8 (38%)	5 (29)
**Subject sleep disorders**		
Epworth sleepiness scale (0-24)	10.57 ± 2.71	10.65 ± 2.57
Berlin questionnaire (items 1-5)	2.29 ± 1.55	2.35 ± 1.73
Berlin questionnaire (items 6-8)	0.76 ± 0.20	0.94 ± 1.03
SBP (mmHg)	119.62 ± 10.26	117.59 ± 7.58
DBP (mmHg)	78.48 ± 6.95	78.29 ± 6.30
RHR (beats/minute)	76.29 ± 11.57	76.29 ± 11.57

Abbreviations: SBP = Systolic blood pressure; DBP = Diastolic blood pressure; RHR = Resting heart rate.

### Outcome variables

EDS was significantly improved after the 6 weeks of training for both the Epworth sleepiness scale (*p*<0.001), effect size=1.16 (Figure 2A), and the Berlin questionnaire (items 6-8) (*p*=0.01), effect size=0.44. Meanwhile, the snoring category (items 1-5) of the Berlin questionnaire showed no differences between baseline and after 6 weeks of training (*p*=0.117) (Figure 2B). The positive risk number in all three categories of the Berlin questionnaire showed a significant decrease after 6 weeks of training (*p*<0.01) (Figure 2C), effect size=0.47. Most of the anthropometric variables showed a significant decrease after 6 weeks of training compared to baseline, including B W, BMI, %BF, HC, and W/H ratio (all *p*<0.05) (Table 2), effect size=0.51-0.64. A significant increase in the estimated VO_2_max (*p*<0.01), (effect size=1.08) was noted after 6 weeks of training (Table 2). RHR showed no significant differences between baseline and at week 6 (*p*=0.074).

**Figure 2A:**
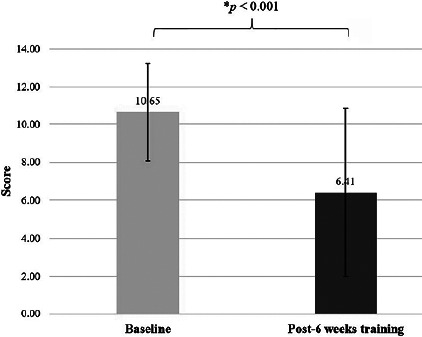
Epworth Sleepiness Scale (* A statistically significant difference between baseline and post-6 weeks of training using Paired sample t-test, p < 0.001).

**Figure 2B:**
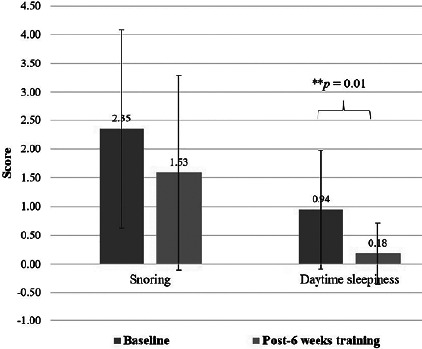
Berlin Questionnaire (** A statistically significant difference between baseline and post-6 weeks of training using Wilcoxon signed rank test, p = 0.01).

**Figure 2C:**
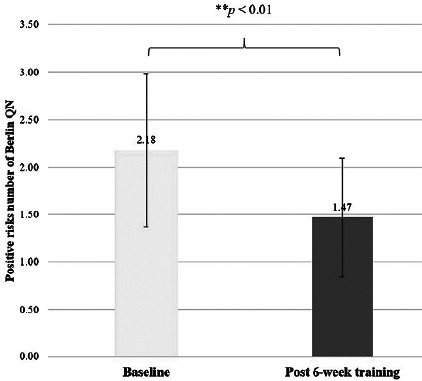
Positive risk numbers of all 3 Berlin questionnaire categories (** A statistically significant difference between baseline and post-6 weeks of training using Wilcoxon signed rank test, p < 0.01).

**Table 2:** Comparison of outcome measurements at baseline and at post-6 weeks of training (n=17).

	Baseline	Post-6 weeks	Mean difference (95%CI)	p
**Anthropometric variables**				
BW (kg)	83.75 ± 15.28	82.51 ± 15.58	− 1.24 (− 2.29 to −0.18)	0.025
BMI (kg/m^2^)	29.42 ± 3.52	28.96 ± 3.59	− 0.46 (− 0.83 to −0.09)	0.018
%BF*	29.00 (26.45-36.60)	28.10 (25.90-35.00)	− 0.86 (− 1.32 to −0.40)	< 0.01
FFM (kg)	58.10 ± 12.22	57.92 ± 12.33	− 0.18 (− 0.73 to 0.37)	0.493
Muscle mass (kg)	55.01 ± 11.72	54.86 ± 11.80	− 0.18 (− 0.69 to −0.34)	0.475
NC (cm)	37.15 ± 2.58	37.06 ± 3.25	− 0.01 (− 1.26 to 1.08)	0.875
WC (cm)	88.85 ± 9.55	90.21 ± 9.31	1.35 (− 0.75 to 3.45)	0.190
HC (cm)*	98.00 (94.00-99.00)	96.00 (92.25-97.50)	− 3.32 (− 4.95 to −1.69)	< 0.001
W/H ratio	0.85 ± 0.06	0.90 ± 0.07	0.05 (0.01 to 0.10)	0.026
**Estimated VO_2_max (ml/kg/min)**	31.40 ± 4.88	37.77 ± 7.63	6.37 (3.35 to 9.38)	< 0.001
**RHR (b/min)**	72.24 ± 11.60	69.76 ± 8.77	− 4.47 (− 9.43 to 0.49)	0.074
**Muscle strength (1-RM, kg)**				
Biceps curl (kg)*	12.00 (7.00-14.50)	14.00 (9.00-15.00)	1.82 (0.97 to 2.68)	0.001
Lateral pull-down (kg)	56.35 ± 11.90	64.82 ± 15.95	8.47 (4.75 to 12.19)	< 0.001
Push-up (kg)	105.18 ± 18.68	117.88 ± 29.53	12.71 (3.07 to 22.34)	0.013
Chest press (kg)*	34.00 (20.50-44.50)	44.00 (33.00-54.50)	10.59 (8.17 to 13.01)	< 0.001
Abdominal crunch (kg)	110.71 ± 16.13	132.76 ± 23.71	22.06 (13.66 to 30.45)	< 0.001
Leg press (kg)	85.59 ± 22.03	99.00 ± 23.96	13.41 (5.19 to 21.63)	0.003
Leg curl (kg)*	14.00 (12.00-16.00)	20.00 (17.00-22.50)	6.06 (3.62 to 8.49)	0.001
**Blood biomarkers**				
TC (mg/dL)	236.35 ± 43.08	230.12 ± 40.43	− 6.24 (to 15.14-2.67)	0.157
Triglyceride (mg/dL)	130.06 ± 55.69	135.24 ± 57.08	5.18 (− 23.85 to 34.20)	0.710
LDL-C (mg/dL)	154.59 ± 43.88	146.88 ± 38.66	− 7.71 (− 19.06 to 3.64)	0.169
HDL-C (mg/dL)	58.88 ± 12.40	56.12 ± 11.04	− 2.76 (− 5.62 to 0.10)	0.057
FBG (mg/dL)*	96.00 (90.50-97.00)	91.00 (89.50-97.50)	− 8.88 (− 14.32 to −3.45)	0.003

Abbreviations: BW = Body weight; BMI = Body mass index; %BF = Percent of body fat; FFM = Fat free mass; NC = Neck circumference; WC = Waist circumference; HC = Hip circumference; W/H = Waist/hip ratio; RHR = Resting heart rate; 1-RM = 1-repetition maximum; TC = Total cholesterol; LDL-C = Low density lipoprotein cholesterol; HDL-C = High density lipoprotein cholesterol; FBG = Fasting blood glucose;

* Wilcoxon signed rank test comparison [data presented as median (IQR)].

The strength measured using 1-RM in all seven poses were significantly higher at week 6 compared to baseline (all *p*<0.05) (Table 2), effect size=0.56-1.35. There were no significant differences for most of the blood biomarkers (all *p*>0.05), except for FBG which showed a significant decrease at week 6 compared to baseline (*p*<0.01) (Table 2), effect size=0.51. There was significant correlation between the data change of ESS to data change of BW, BMI, FFM, and muscle mass (*p*=0.004, *p*=0.005, *p*=0.010, and *p*=0.015, respectively). There was a significant correlation between the change score of Berlin QN (snoring category) to the change score of muscle strength (chest press) (*p*=0.008) (Table 3).

**Table 3:** Correlations between data change of the subjective sleep disorders to anthropometric variables, physical fitness, and FBG (n=17).

	Epworth sleepiness scale		Berlin QN (Snoring category, items 1-5)		Berlin QN (Daytime sleepiness category, items 6-8)	
	Spearman correlation	p	Spearman correlation	p	Spearman correlation	p
BW (kg)	0.66 a	0.004	0.16	0.546	0.073	0.779
BMI (kg/m2)	0.64 a	0.005	0.17	0.512	0.08	0.759
%BF	0.30	0.241	0.02	0.943	0.27	0.302
FFM (kg)	0.60 a	0.010	0.01	0.966	− 0.08	0.759
Muscle mass (kg)	0.58 a	0.015	0.09	0.727	− 0.05	0.837
NC (cm)	− 0.00	0.994	0.07	0.803	0.46	0.063
WC (cm)	0.354	0.163	− 0.14	0.592	− 0.31	0.228
HC (cm)	0.47	0.055	0.04	0.880	0.16	0.544
Estimated VO_2_max (ml/kg/min)	− 0.30	0.247	0.15	0.576	− 0.33	0.899
**Muscle strength (kg)**						
Biceps curl	− 0.31	0.237	0.02	0.950	0.21	0.425
Lateral pull-down	− 0.31	0.229	0.04	0.892	0.19	0.470
Push-up	0.14	0.597	0.09	0.731	− 0.24	0.346
Chest press	0.03	0.906	0.62 a	0.008	0.28	0.283
Abdominal crunch	0.32	0.205	− 0.19	0.462	− 0.14	0.595
Leg press	0.04	0.891	0.08	0.754	0.00	1.000
Leg curl	0.04	0.894	0.31	0.225	0.45	0.069
FBG (mg/dL)	− 0.15	0.555	0.13	0.621	− 0.35	0.173

Abbreviations: BW = Body weight; BMI = Body mass index; %BF = Percent of body fat; FFM = Fat free mass; NC = Neck circumference; WC = Waist circumference; HC = Hip circumference; FBG = Fasting blood glucose;

a Correlation is significant at p-value < 0.05 (two-tailed).

## DISCUSSION

This study was the first to determine the effects of a short period of HIIT combined with RT on the subjective sleep problem in adults with obesity and SRBDs. The main findings revealed that 6 weeks of this combination program was able to ameliorate EDS, lower the risk of SRBDs, improve most of the anthropometric variables, and increase physical fitness in adults with obesity and SRBDs. These results are similar to previous studies which showed an improvement in sleep quality^7,10,11,12,25,26,27^, and sleep indices^12,25,28^ after completion of exercise programs. However, the study period for those studies was longer than the present study, excepted for the study of Ebrahimi et al. (2017)^27^ which examined the effect of yoga and aerobic exercise at week 6 and week 12. In addition, the exercise prescription of those previous studies was performed differently to our study. For example, some studies used individual aerobic exercises (AE)^7^, AE plus RT^12^, diet and AE combined with RT^10^, home-based combination program for the aging^11^, and lifestyle modification including diet and physical activity^7,29^. There were limited studies using a HIIT program for SRBDs patients^19,20^. There was only one study examining the effects of the HIIT program in adults with obesity, where the results showed an improvement in sleep indices and EDS^19^. Even though our study did not assess sleep indices using polysomnography, there was a study show the association between the sleep indices and most of the subjective sleep questionnaires^21,22^. Therefore, the reduction of both EDS and positive risk numbers in all three Berlin questionnaire categories may indicate an improvement in sleep indices.

The present study revealed a reduction in the anthropometric variables including B W, %BF, HC, and W/H ratio. There is an evidence supported that these anthropometric variables are positively correlated to OSA severity^30^. Our results support this notation which found correlation between the change score of BW and BMI to the Epworth sleepiness scale. Therefore, BW and BMI are the cornerstone for the improvement of subjective sleep disorders in our study. However, there are studies which show that OSA severity was achieved without a significant reduction in body composition, which is different to our findings^25,28^. Additionally, increasing BW is also positively related to inflammatory markers and oxidative stress^31^. These cytokines result in airway collapse^32^. Hence, the effects of HIIT plus RT might cause an increase in anti-inflammatory cytokines and antioxidant agents^33^. Unfortunately, only blood lipid profiles and FBG were examined in the present study and no changes were found except for FBG. It might be inferred that a 6-week exercise period is insufficient to show a significant improvement in blood biomarkers. Noticeably, most of the blood biomarkers were in the normal range; thus, the ceiling for improvement was limited. In addition, the diet program of participants was not controlled in our study compared to a previous study^10^. This study showed an improvement in most of the blood biomarkers including insulin levels, triglyceride, and C-reactive protein after an intensive 16 weeks of AE combined with RT and a controlled diet^10^. Therefore, HIIT combine with RT plus dietary control might be another program challenge to prescribe for adults with obesity and SRBDs.

The estimated VO_2_max and muscle strength in this study showed an apparent increase after 6 weeks of training. Our exercise program may consequently increase respiratory muscle strength, lung compliance, ventilation/perfusion matching, and vascular structure and function, which induces an improvement in intermittent hypoxemia during sleep^34^. Also, a reduction in the accumulation of abdominal visceral fat and adipose tissue at the chest wall might be another factor in improving breathing during sleep^35^. Interestingly, among of the change score of muscle strengths, there was only chest muscle was found to significantly correlate with the Berlin questionnaire (items 1-5) (*p*=0.008). Therefore, chest muscle strength may involve in an improvement of the sleep quality such as snoring. The findings of this study were consistent with many previous studies which showed an improvement in VO_2_max^10,19,28^ and muscle strength^10^ after completion of an exercise intervention program. However, measurement methods and exercise periods were different compared to our study. The percent of change for VO_2_max in our study was greater than in a previous study^19^. This might be caused by the participants in our study were identified as sedentary persons with obesity who were younger than participants in the previous study^19^, and the severity of SRBDs in our participants was lower than the previous study^19^.

Even though the findings in the present study showed positive effects in most of the variables, there were some limitations observed including: 1) this study was a one group design and the next study should be a comparable study design, such as a randomized controlled usual care group; 2) the dietary of participants were not examined in this study, thus the amount of food intake should be monitored to confirm the findings; 3) inflammatory and oxidative biomarkers should be examined in a future study, and therefore the mechanism behind the improvement of sleep quality might be better explained than in the present study; and 4) the severity of SRBDs in most previous studies were moderate to severe which may be different to the present study, and therefore changes in higher degrees of SRBD severity might be easier to observe than in our study. However, the strength of this study indicated that 6 weeks of HIIT combined with RT was feasible and could improve the subjective sleep disorders in adults with obesity. These improvements are ranged in moderate to large effect size even though there were limited in sample size.

In conclusion, six weeks of HIIT combined with RT is safe and can improve subjective sleep disorders, anthropometric variables, and physical fitness. Therefore, these combined programs may be beneficial for the remedy of subjective sleep disorders in adults with obesity and SRBDs.
